# Chicken gga-miR-103-3p Targets CCNE1 and TFDP2 and Inhibits MDCC-MSB1 Cell Migration

**DOI:** 10.1534/g3.116.028498

**Published:** 2016-03-01

**Authors:** Bo Han, Ling Lian, Xin Li, Chunfang Zhao, Lujiang Qu, Changjun Liu, Jiuzhou Song, Ning Yang

**Affiliations:** * Department of Animal Genetics and Breeding, National Engineering Laboratory for Animal Breeding, College of Animal Science and Technology, China Agricultural University, Beijing 100193, China; † Division of Avian Infectious Diseases, Harbin Veterinary Research Institute of Chinese Academy of Agricultural Sciences, Harbin 150001, China; ‡ Department of Animal and Avian Sciences, University of Maryland, College Park, Maryland 20742

**Keywords:** chicken, Marek’s disease, gga-miR-103-3p, *CCNE1*, *TFDP2*, cell migration

## Abstract

Marek’s disease (MD) is a highly contagious viral neoplastic disease caused by Marek’s disease virus (MDV), which can lead to huge economic losses in the poultry industry. Recently, microRNAs (miRNAs) have been found in various cancers and tumors. In recent years, 994 mature miRNAs have been identified through deep sequencing in chickens, but only a few miRNAs have been investigated further in terms of their function. Previously, gga-miR-103-3p was found downregulated in MDV-infected samples by using Solexa deep sequencing. In this study, we further verified the expression of gga-miR-103-3p among MDV-infected spleen, MD lymphoma from liver, noninfected spleen, and noninfected liver, by qPCR. The results showed that the expression of gga-miR-103-3p was decreased in MDV-infected tissues, which was consistent with our previous study. Furthermore, two target genes of gga-miR-103-3p, cyclin E1 (*CCNE1*) and transcription factor Dp-2 (E2F dimerization partner 2) (*TFDP2*), were predicted and validated by luciferase reporter assay, qPCR, and western blot analysis. The results suggested that *CCNE1* and *TFDP2* are direct targets of gga-miR-103-3p in chickens. Subsequent cell proliferation and migration assay showed that gga-miR-103-3p suppressed MDCC-MSB1 migration, but did not obviously modulate MDCC-MSB1 cell proliferation. In conclusion, gga-miR-103-3p targets the *CCNE1* and *TFDP2* genes, and suppresses cell migration, which indicates that it might play an important role in MD tumor transformation.

Marek’s disease (MD) is a highly contagious T-cell lymphoid neoplasia of chickens induced by an alphaherpesvirus, gallid herpesvirus type 2 (GaHV-2), which is historically known as Marek’s disease virus (MDV) (Gennart *et al.* 2015). MDV infection can be generally divided into four phases: early cytolytic, latent, late cytolytic, and tumor transformation phase (Calnek 2001). The disease is characterized by monocyte infiltration, and the formation of tumor lesions in chickens peripheral nerves, gonad, iris, various organs, muscles, skin, and other tissues (Calnek 1986, 2001), which causes huge economic losses to the poultry industry. Currently, MD can be prevented by virus vaccine, which is injected into 1-d-old chickens. However, the efficacy of the vaccine has decreased concomitantly with the increase in virulence of MDV (Gimeno 2008). Therefore, it is important to explore better approaches to control this disease, for which investigating the mechanism of resistance, and the susceptibility of chickens, will be among the most important strategies.

MicroRNAs (miRNAs) are small, noncoding, endogenous single-strand RNA molecules of 21–26 nt, which repress gene expression by binding to complementary sequences in the 3ʹ-untranslated region (3ʹ-UTR) of mRNAs to prevent their translation (Bartel 2009). miRNAs are also involved in multiple cellular processes, including cell proliferation, migration, and apoptosis (Wang *et al.* 2010). Extensive studies on miRNAs have been carried out in human cancers (Calin and Croce 2006; Farazi *et al.* 2013; Iorio and Croce 2012; Lu *et al.* 2005). In chickens, miRNAs have been studied, and the roles they play in growth performance, reproduction, and disease resistance have been elucidated (Burnside and Morgan 2011; Wang *et al.* 2013; Han *et al.* 2015; Li *et al.* 2012, 2013; Lin *et al.* 2012; Kang *et al.* 2013; Rengaraj *et al.* 2013; X. Wang *et al.* 2014; Zhang *et al.* 2013). The chicken and MDV-encoded miRNAs were first identified in MDV-infected chicken embryo fibroblasts (CEF) by Burnside *et al.* (2006). Since then, many viral miRNAs had been found, and are considered to play critical roles in MDV tumorigenesis (Morgan *et al.* 2008; Zhao *et al.* 2009, 2011; Luo *et al.* 2011; Xu *et al.* 2011; Hicks and Liu 2013; Z. Li *et al.* 2014; Yu *et al.* 2014; Chi *et al.* 2015; Teng *et al.* 2015). However, the functions of host miRNAs involved in chicken MD still need to be investigated (Burnside *et al.* 2008; Lian *et al.* 2012a, 2012b, 2015; Tian *et al.* 2012; X. Li *et al.* 2014).

In a previous study (Lian *et al.* 2012a), we identified 187 known chicken miRNAs in MDV-infected samples by Solexa deep sequencing, among which, gga-miR-103-3p was downregulated in MDV-infected groups. In this study, we further verified its targets and conducted a preliminarily investigation into the role of gga-miR-103-3p in MDCC-MSB1 cell proliferation and migration.

## Materials and Methods

### Ethics statements

Animal experiments were specifically approved by the Animal Care and Use Committee of China Agricultural University (Approval ID: XXCB-20090209), and this study was carried out in strict accordance with the regulations and guidelines established by this committee. The animals were killed using T61 intravenously before dissection for sample collection. All efforts were made to minimize suffering.

### Sample collection and experimental design

In our previous study, MDV challenge trial was conducted with 150 chickens from a White Leghorn specific pathogen-free line (BWEL) (Lian *et al.* 2010). A total of 100 1-d-old chickens was infected intraperitoneally with 2000 PFU of the MDV GA strain in CEF, and 50 chickens were injected with the same dosage of CEF cells (0.2 ml) as controls. The two groups were housed in separate isolators. The chickens were observed until 56 d postinfection (dpi). During this phase, the clinical signs of infected chickens were observed two to three times daily. Some chickens showed severe appetite loss, serious distress and depression, and impending death. These severely morbid chickens, and one to three age-matched noninfected chickens were killed using T61 intravenously (0.3 ml/kg) (Intervet, Ukkel, Belgium). The dead chickens were dissected, and their whole spleens and MD lymphoma in livers were collected. Eight MDV-infected tumorous spleens, eight MD lymphomas from liver, eight noninfected spleens, and eight noninfected livers collected during 30–56 dpi (Lian *et al.* 2012a) were used in this study. A schematic of the experimental design is shown in Supplemental Material, Figure S1. The binding sites of gga-miR-103-3p in the 3ʹ-UTR of target genes were confirmed by luciferase reporter assay, and the expression of target genes in mRNA, and protein levels, were validated using real-time PCR and western blot analysis, respectively; moreover, the biological function of gga-miR-103-3p in cell proliferation and migration was investigated.

### RNA isolation and real-time PCR for quantifying gga-miR-103-3p expression in tissue

Total RNA was isolated from tissue samples using a mirVana miRNA Isolation Kit (Ambion, Life Technologies, Carlsbad, CA), as described by the manufacturer, and reverse transcribed using a miRACLE cDNA Synthesis Kit (Genetimes Technology, Shanghai, China). Real-time PCR was performed on an ABI 7500 system (Applied Biosystems, Foster City, CA). A specific forward primer, and universal reverse primer, for gga-miR-103-3p, and a reference small noncoding RNA (chicken 5S rRNA), were designed and synthesized by Genetimes Technology, Inc (Table 1). Real-time PCR was performed using miRACLE qPCR miRNA Master Mix kit (Genetimes Technology). The optimum thermal cycling parameters were 95° for 10 min, 40 cycles of 95° for 10 s, 60° for 20 s, 72° for 1 min, 95° for 15 s, 60° for 30 s, and 95° for 15 s. All experiments were run in triplicate. The expression of gga-miR-103-3p was measured using the 2^–ΔΔCt^ method.

**Table 1:** Real-time PCR primers for detecting expression of gga-miR-103-3p

miRNA	Direction*^a^*	Sequence
gga-miR-103-3p	Forward	AGCAGCATTGTACAGGGCTATGAA
Chicken 5S rRNA*^b^*	Forward	ACCGGGTGCTGTAGGCTTAA

a The universal reverse primer was provided by the kit of miRACLE qPCR miRNA Master Mix (Genetimes Technology, Shanghai, China).

b 5S rRNA acted as a reference.

### Target genes prediction

The target genes of gga-miR-103-3p were predicted using the online tools TargetScan (http://www.targetscan.org) and miRDB (http://mirdb.org/miRDB/). Gene ontology (GO) analysis was conducted to screen the genes related to disease, immune system, cell cycle, cell proliferation, and cell migration by DAVID (Database for Annotation, Visualization and Integrated Discovery, http://david.abcc.ncifcrf.gov/).

### Luciferase reporter assay

The human embryonic kidney 293T (HEK 293T) cell line was used to perform the luciferase reporter assay. Cells were grown in Dulbecco’s modified Eagle’s medium (DMEM) (Gibco, Life Technologies, Carlsbad, CA) supplemented with 10% fetal bovine serum (FBS) (Gibco). All cells were maintained at 37° in a humidified incubator containing 5% CO_2_. The luciferase reporter vectors were constructed (RiboBio, Guangzhou, China). Briefly, fragments of 3ʹ-UTR sequences covering the putative gga-miR-103-3p binding sites were amplified, and then inserted into pmiR-RB-REPORT luciferase reporter vectors to construct wild-type 3ʹ-UTR vector. Mutant 3ʹ-UTR vector was constructed by PCR amplification with wild-type 3ʹ-UTR vector as template. The primers for wild-type (wt) and mutant-type (mut) vector were shown in Table 2. The *CCNE1* wt UTR reporter and three mutant-type 3ʹ-UTR reporters corresponding to three potential binding sites, including *CCNE1* mut (75–81) UTR, *CCNE1* mut (248-254) UTR, and *CCNE1* mut (475-481) UTR, were constructed. Two wild luciferase reporters for *TFDP2* gene were constructed because of the long sequence of its 3ʹ-UTR region: *TFDP2* wt (1–1252) UTR and *TFDP2* wt (5161–6415) UTR. These two reporters have one and two binding sites for gga-miR-103-3p, respectively. These three reporters, *TFDP2*(1–1252) mut (28–34) UTR, *TFDP2* (5161–6415) mut (5609–5615) UTR, and *TFDP2* (5161–6415) mut (6254-6260) UTR, were constructed for detecting the real binding site. The gga-miR-103-3p mimics and mimics negative control (NC) were synthesized by GenePharma (Shanghai, China; Table 3). HEK 293T cells were plated into 96-well plates, and cultured 24 hr before cotransfection at 60% confluence. Then, pmiR-RB-Report luciferase reported vector containing wild type or mutant type were cotransfected with gga-miR-103-3p mimics, or NC, into HEK 293T cells using FuGENE HD transfection reagent (Promega, Madison, WI). Luciferase activity was measured 24 hr after cotransfection by the Dual-Luciferase Reporter Assay System (Promega) following the manufacturer’s instructions. The results were expressed as relative luciferase activities (Renilla luciferase/Firefly luciferase) normalized against the mimics NC group. All experiments were performed in triplicate.

**Table 2:** Primers for wild-type and mutant-type vector construction

Gene and Type	Direction	Sequence
*CCNE1* wtUTR	Forward	CCGCTCGAGCTGTACGAACTGTTTACAG
	Reverse	TAAGCGGCCGCAAAGTATACGCCAAAATC
*CCNE1* mut(75–81) UTR*^a^*	Forward	TTATGGAATACGACGCGGTGACATTCTAAAGCT
	Reverse	TGTCACCGCGTCGTATTCCATAATGGAGCAGGA
*CCNE1* mut(248–254) UTR*^a^*	Forward	TGCTCCAATACGACGTATTGAGGGTGATGCTTG
	Reverse	CCTCAATACGTCGTATTGGAGCACTCTTCGGTG
*CCNE1* mut(475–481) UTR*^a^*	Forward	TATTGTAATACGACGTATGTCTCTGTGTATCCA
	Reverse	GAGACATACGTCGTATTACAATAAAGAGTTTTT
*TFDP2*(1–1252) wtUTR*^b^*	Forward	CCGGCTCGAGAGACAGTGAAAAAAATGGATAC
	Reverse	TAAGCGGCCGCTATGAGAACCGTACATCTAC
*TFDP2*(1–1252)*^b^* mut(28–34) UTR*^a^*	Forward	AGACAGTGAAAAAAATGGATACACAAATACGACGACATATATATTCTTAATGG
	Reverse	TAAGCGGCCGCTATGAGAACCGTACATCTAC
*TFDP2*(5161–6415) wtUTR*^b^*	Forward	CCGGCTCGAGAGACAGTGAAAAAAATGGATAC
	Reverse	TAAGCGGCCGCTATGAGAACCGTACATCTAC
*TFDP2*(5161–6415)*^b^* mut(5609–5615) UTR*^a^*	Forward	ACTTTTTATACGACGACTTAAAGTTTGTAAACTT
	Reverse	ACTTTAAGTCGTCGTATAAAAAGTAATGTTTTTG
*TFDP2*(5161–6415)*^b^* mut(6254–6260) UTR*^a^*	Forward	TACTGAAATACGACGAAGCGGGAATTGTACATTG
	Reverse	TTCCCGCTTCGTCGTATTTCAGTAAACATGGAAT

wt, wild type; mut, mutant.

a Mutation position in 3ʹ-UTR.

b Position in 3ʹ-UTR.

**Table 3:** Sequences of gga-miR-103-3p mimics, mimics negative control (NC), inhibitor, and inhibitor NC (GenePharma, Shanghai, China)

Name	Strand	Sequence (5ʹ–3ʹ)
gga-miR-103-3p mimics	Sense	AGCAGCAUUGUACAGGGCUAUGA
	Antisense	AUAGCCCUGUACAAUGCUGCUUU
Mimics negative control (NC)	Sense	UUCUCCGAACGUGUCACGUTT
	Antisense	ACGUGACACGUUCGGAGAATT
gga-miR-103-3p inhibitor	—	UCAUAGCCCUGUACAAUGCUGCU
Inhibitor negative control (NC)	—	CAGUACUUUUGUGUAGUACAA

### Gain/loss function study

The MD lymphoma cell line MDCC-MSB1 [provided by Dr. C. Itakura (Akiyama and Kato 1974; Goryo *et al.* 1987)] was cultured in RPMI-1640 medium (Gibco) with 10% FBS. All cells were maintained at 37° in a humidified incubator containing 5% CO_2_. The gga-miR-103-3p mimics, mimics NC, gga-miR-103-3p inhibitor, and inhibitor NC were transfected into MDCC-MSB1 cells with FuGENE HD transfection reagent, respectively. Total RNA was isolated at 24 hr, 48 hr, and 72 hr after transfection, and real-time PCR was performed as described above for validating the expression of gga-miR-103-3p. To detect gene expression, total RNA was extracted from MDCC-MSB1 cells using E.Z.N.A. Total RNA Kit II (Omega, GA), and reverse transcribed into cDNA by EasyScript First-Strand cDNA Synthesis SuperMix (TransGen Biotech, Beijing, China). Each reaction was in a final volume of 15 μl, containing 1 μl of cDNA, 0.2 μl of each gene primer (100 nM, Table 4), and 1× PCR mix (Power SYBR Green PCR Master Mix, Applied Biosystems), and the optimum thermal cycling parameters included 95° for 10 min, 40 cycles of 95° for 10 s, 60° for 1 min, 95° for 15 s, 60° for 30 s, and 95° for 15 s. All experiments were run in triplicate. The expression of gga-miR-103-3p, and the two targets, were measured using the 2^–ΔΔCt^ method.

**Table 4:** Real-time PCR primers for target genes

Gene	Direction	Sequence
*CCNE1*	Forward	CTGCTGGTTCTAACTCCTGCTC
	Reverse	TGGCGTACTCGATCCATTTCTAT
*TFDP2*	Forward	CCCAGCATCAAATTCCACAA
	Reverse	GCCTTCCTCAAGCCCAAAG
*GAPDH**^a^*	Forward	GAAGCTTACTGGAATGGCTTTCC
	Reverse	GGCAGGTCAGGTCAACAACAG

a Reference gene.

### Western blot

MDCC-MSB1 cells were seeded into six-well plates, and transfected with gga-miR-103-3p mimics, mimics NC, gga-miR-103-3p inhibitor, and inhibitor NC, respectively. Proteins were isolated at 72 hr post transfection using radio immunoprecipitation assay (RIPA) lysis buffer with phenylmethanesulfonylfluoride (PMSF). The concentration of proteins was determined by the BAC assay (BCA Protein Assay Kit, Beyotime, Shanghai, China). Western blot was performed using standard methods (Blake *et al.* 1984; Dennis-Sykes *et al.* 1985). Proteins and protein marker (PageRuler Prestained Protein Ladder, Thermo Scientific, Life Technologies, Carlsbad, CA) were separated by 10% SDS-PAGE gels, and then transferred to polyvinylidene difluoride (PVDF) membranes. Membranes were blocked for 1 hr, and incubated overnight with Cyclin E Antibody (Novus Biologicals, Littleton, CO, 1:1000), Rabbit Anti-*TFDP2* Polyclonal Antibody (Bioss Antibodies, Woburn, MA, 1:1000), and Actin antibody (Bayotime, Shanghai, China, 1:1000). After washing, the membranes were incubated for 1 hr with HRP-labeled Goat Anti-Rabbit IgG (H+L) (1:1000), HRP-labeled Goat Anti-Rabbit IgG (H+L) (1:1000), and HRP-labeled Goat Anti-Mouse IgG (H+L) (1:1000), respectively (Bayotime). Proteins were detected using BeyoECL Plus (Bayotime). The grayscale values of protein bands were analyzed using ImageJ. The grayscale value of *CCNE1* or *TFDP2* was standardized to that of *ACTIN*.

### Cell proliferation and migration assay

A total of 1 × 10^4^ MDCC-MSB1 cells per well were seeded into 96-well plates, and transfected with gga-miR-103-3p mimics, mimics NC, gga-miR-103-3p inhibitor, and inhibitor NC, respectively. The CCK-8 (Cell Counting Kit-8, Beyotime) was added to each well at 24 hr, 48 hr, and 72 hr post transfection, and incubated at 37° for 2 hr. The absorbance at 450 nm was measured using the microplate spectrophotometer. All experiments were performed in triplicate.

The migration ability of MDCC-MSB1 cells was detected using Transwells (8 mm pore size, Corning Costar Corp, Corning, NY) at 48 hr after transfection. Transwells were put into the 24-well plates. A total of 1.5 × 10^4^ cells/well were seeded in the upper chamber, and 500 μl of RPMI-1640 containing 20% FBS was added into the lower chambers. After incubating for 16 hr at 37° in a 5% CO_2_ humidified incubator, the cells were counted under a microscope in three independent fields, with magnification of × 100. The mean of triplicate assays for each experimental condition was used. All experiments were performed in triplicate.

### Data analysis

Data were expressed as the mean ± SD. The data were analyzed using a two-tailed Student’s *t*-test, and the differences were considered to be statistically significant at *P* < 0.05.

### Data availability

File S1 contains the target genes of gga-miR-103-3p predicted by TargetScan and miRDB, and clustered by GO analysis.

## Results

### Differential expression of gga-miR-103-3p between MDV-infected and noninfected groups

**Figure 1:**
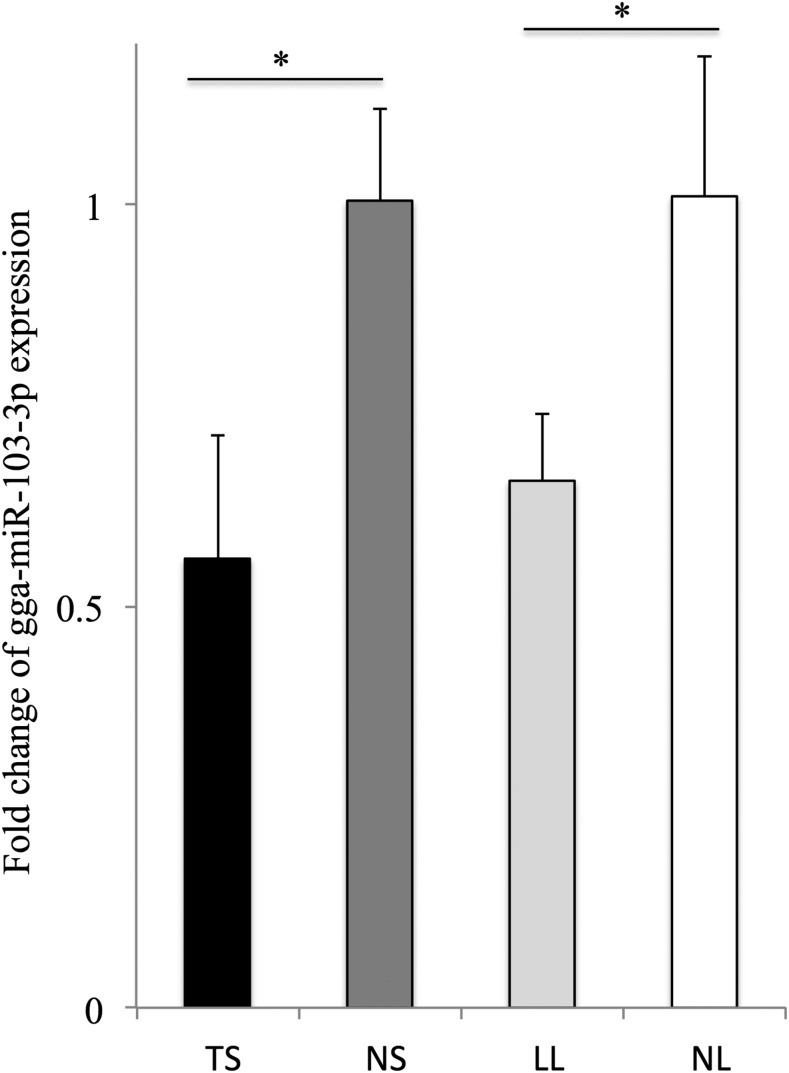
Differential expression of gga-miR-103-3p among tumorous spleen, noninfected spleen, MD lymphoma from liver, and noninfected liver. Results were measured using the 2^–ΔΔCt^ method. The fold change of TS was compared with that of NS, and NL was the control for LL. * *P* < 0.05. TS, tumorous spleen; NS, noninfected spleen; LL, MD lymphoma from liver; NL, noninfected liver.

The expression of gga-miR-103-3p among tumorous spleen, MD lymphoma from liver, noninfected spleen, and noninfected liver, was detected by qPCR. The results showed that expression of gga-miR-103-3p was significantly downregulated in tumorous spleen and MD lymphoma from liver compared with the noninfected tissues (Figure 1).

### Prediction and verification of gga-miR-103-3p target genes

In total, 236 and 316 target genes were predicted by TargetScan and miRDB, respectively. Fifty-six genes were predicted in both databases, which were used for GO analysis on the DAVID platform. The 56 common genes were clustered to different kinds of biological processes, including energy metabolism, ion transport, and cellular processes. Cyclin E1 (*CCNE1*), and transcription factor DP-2 (E2F dimerization partner 2) (*TFDP2*), the two genes with the highest prediction score in both TargetScan and miRDB, are linked with the cell cycle (KEGG Pathway: map 04110), and TFDP2 is involved in regulation of transcription (GO:0045449) (File S1). Referring to the results of GO analysis, and our previous microarray study (Lian *et al.* 2012a), *CCNE1* and *TFDP2* were chosen for further validation.

**Figure 2:**
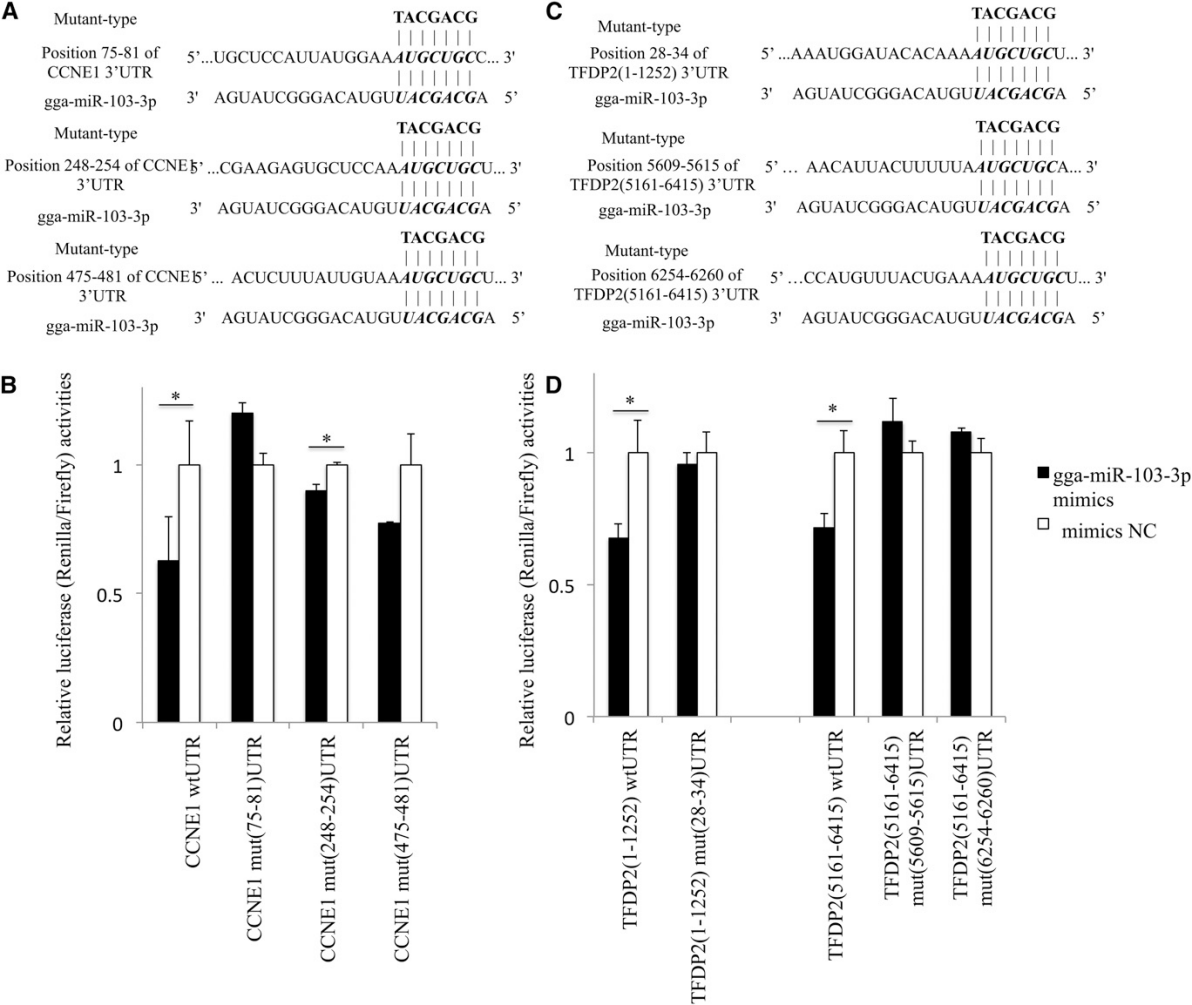
Luciferase reporter assay. (A) Nucleotide sequences of *CCNE1* wild type and mutant in the 3ʹ-untranslated region (3ʹ-UTR). Three potential binding sites were marked in bold italic. (B) The relative luciferase activities of *CCNE1* wild type and mutant reporters normalized against mimics NC. (C) Nucleotide sequences of *TFDP2* wild type and mutant in the 3ʹ-UTR. Three potential binding sites were marked with bold italic. (D) The relative luciferase activities of *TFDP2* wild and mutant reporters normalized against mimics NC. Two wild luciferase reporters [*TFDP2* wt (1–1252) UTR and *TFDP2* wt (5161–6415) UTR] for the *TFDP2* gene were constructed because of its long 3ʹ-UTR region. * *P* < 0.05.

The luciferase reporter system was used to detect the interaction of miRNA and its predicted target genes. The results showed that relative luciferase activity of *CCNE1* wt UTR reporter was significantly inhibited by 40% when cotransfected with gga-miR-103-3p mimics compared with mimics NC (Figure 2B). The three mutant-type reporters, including *CCNE1* mut (75–81) UTR, *CCNE1* mut (248–254) UTR, and *CCNE1* mut (475–481) UTR reporter, were cotransfected with gga-miR-103-3p mimics, and mimics NC, respectively. The results showed that the relative luciferase activities of *CCNE1* mut (248–254) UTR reporter transfected with gga-miR-103-3p mimics was significantly decreased compared with mimic NC. *CCNE1* mut (75–81) UTR and *CCNE1* mut (475–481) UTR had no significant change, but the relative luciferase activities of *CCNE1* (475–481) UTR displayed a downtrend when this reporter was cotransfected with gga-miR-103-3p mimics (Figure 2B). Taken together, the results demonstrate strongly that the *CCNE1* (248–254) UTR was not the binding site of gga-miR-103-3p. The *CCNE1* (75–81) UTR and *CCNE1* (475–481) UTR could bind gga-miR-103-3p, and the *CCNE1* (75–81) UTR was the primary binding site of gga-miR-103-3p.

The relative luciferase activities of *TFDP2* wt (1–1252) UTR and *TFDP2* wt (5161–6415) UTR reporters cotransfected with gga-miR-103-3p mimics were both significantly decreased (Figure 2D). However, the relative luciferase activities of the three mutant-type luciferase reporters exhibited no significant change after cotransfection (Figure 2D). These results suggest that gga-miR-103-3p could bind to three sites in the target *TFDP2* mRNA. Overall, *CCNE1* and *TFDP2* are the two candidate target genes for gga-miR-103-3p.

**Figure 3:**
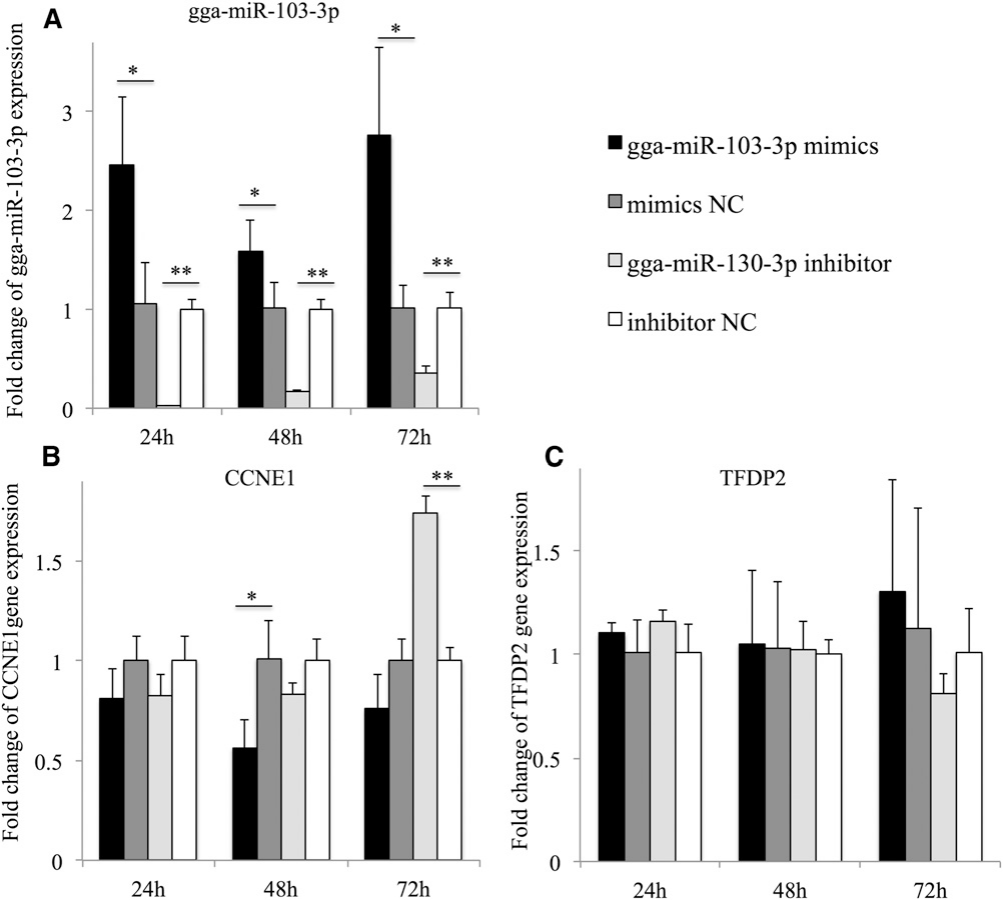
Expression of (A) gga-miR-103-3p, (B) *CCNE1*, and (C) *TFDP2* at 24 hr, 48 hr, and 72 hr. Results showed the fold change of expression using the 2^–ΔΔCt^ method. Chicken 5S rRNA and *GAPDH* were the endogenous reference for gga-miR-103-3p and the two target genes, respectively. The gga-miR-103-3p mimics group was compared with mimics NC group, and inhibitor NC group was the control for gga-miR-103-3p inhibitor group. * *P* < 0.05, ** *P* < 0.01.

The RNA expression of gga-miR-103-3p, *CCNE1*, and *TFDP2* in MDCC-MSB1 cells was examined at 24 hr, 48 hr, and 72 hr after transfecting gga-miR-103-3p mimics, mimics NC, gga-miR-103-3p inhibitor, and inhibitor NC, respectively. As shown in Figure 3, the expression level of gga-miR-103-3p was significantly higher in the gga-miR-103-3p mimics group, and lower in the gga-miR-103-3p inhibitor transfection group than that in corresponding negative control, respectively (Figure 3A). On the contrary, expression of *CCNE1* was significantly lower at 48 hr after transfection with gga-miR-103-3p mimics compared with the mimics NC group. At 24 hr and 72 hr, the expression of *CCNE1* in the mimics groups exhibited no marked change, but there was a visible downward trend. The expression of *CCNE1* at 72 hr after transfecting MDCC-MSB1 cells with gga-miR-103-3p inhibitor was notably increased compared with the inhibitor NC group, which indirectly indicated that gga-miR-103-3p could bind with *CCNE1* (Figure 3B). However, there was no obvious change in *TFDP2* mRNA level after transfection (Figure 3C). The results demonstrated that gga-miR-103-3p could interfere with expression of *CCNE1* mRNA, but did not regulate *TFDP2* mRNA expression.

**Figure 4:**
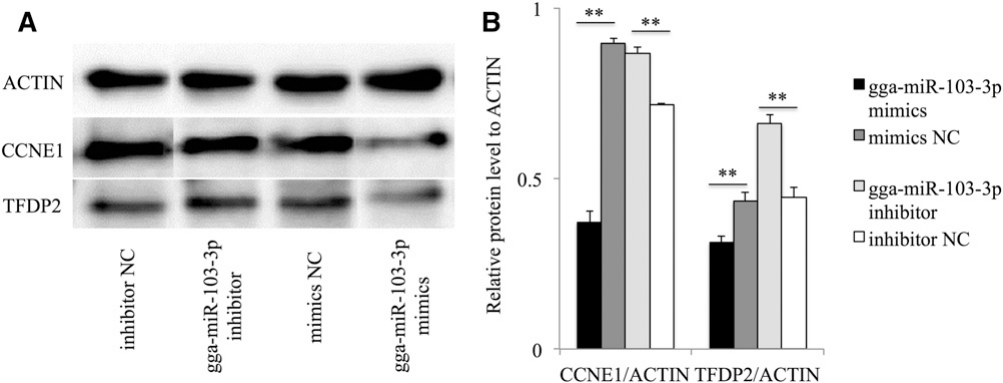
Protein expression levels of *CCNE1*, *TFDP2*, and *ACTIN* at 72 hr in MDCC-MSB1 cells after transfecting gga-miR-103-3p mimics, mimics NC, gga-miR-103-3p inhibitor, and inhibitor NC, respectively. (A) Results of western blot assay. (B) The ratio of gray scale values of *CCNE1* and *TFDP2* to that of *ACTIN*. ** *P* < 0.01.

The protein expression levels of *CCNE1* and *TFDP2* were detected in MDCC-MSB1 cells at 72 hr after transfection with gga-miR-103-3p mimics, mimics NC, gga-miR-103-3p inhibitor, and inhibitor NC, respectively. The results showed that the protein levels of *CCNE1* and *TFDP2* were significantly reduced after transfection with gga-miR-103-3p mimics compared with mimics NC, and the relative protein expression of these two genes in gga-miR-103-3p inhibitor groups were notably increased compared to inhibitor NC groups, respectively (Figure 4). Results of target mRNA expression and western blot assay indicated that gga-miR-103-3p regulated *CCNE1* gene expression at both mRNA and protein levels. However, gga-miR-103-3p could modulate *TFDP2* at the protein but not the mRNA level.

### The effects of gga-miR-103-3p on cell proliferation and migration

**Figure 5:**
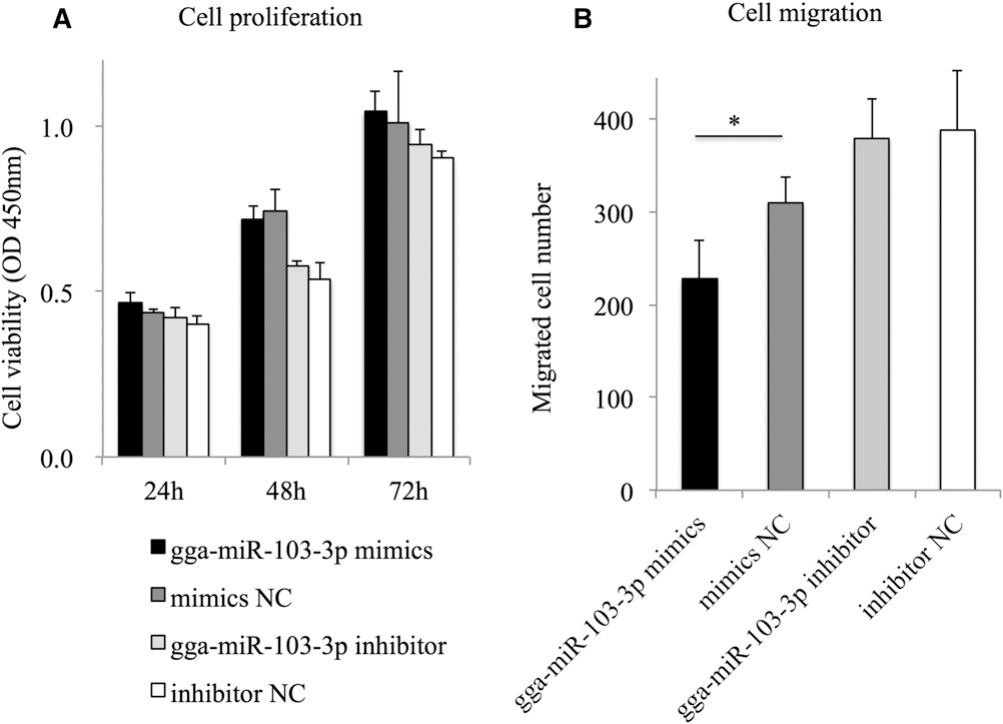
Effects of gga-miR-103-3p on cell proliferation and migration of Marek’s disease lymphoma cell line (MDCC-MSB1) cells. (A) Cell viability of MDCC-MSB1 cells after transfection with gga-miR-103-3p mimics, mimics NC, gga-miR-103-3p inhibitor, and inhibitor NC, respectively. OD, optical density. (B) The number of migrated MDCC-MSB1 cells at 48 hr after transfection with gga-miR-103-3p mimics, mimics NC, gga-miR-103-3p inhibitor, and inhibitor, respectively. Cell number is the mean of triplicate assays for each experimental condition. * *P* < 0.05.

To explore the role played by gga-miR-103-3p in MDCC-MSB1 cells, we performed cell proliferation and migration assays after overexpressing or interfering with gga-miR-103-3p. The increasing cell viability at various time points indicated that cell proliferation increased over time. However, the cell viability exhibited no obvious change at 24 hr, 48 hr, and 72 hr in gga-miR-103-3p mimics or gga-miR-103-3p inhibitor groups, compared with the corresponding NC group (Figure 5A). This suggested that gga-miR-103-3p could not influence MDCC-MSB1 cell proliferation. As shown in Figure 5B, the migrated cell number in the gga-miR-103-3p mimics transfection group was notably decreased compared with the mimics NC group, suggesting that overexpression of gga-miR-103-3p could inhibit MDCC-MSB1 cell migration at 48 h. Collectively, gga-miR-103-3p could suppress MDCC-MSB1 cell migration, but has no obvious effects on its proliferation.

## Discussion

MicroRNAs are associated with a variety of diseases, including cancers and oncosis (Chen *et al.* 2008). It was reported that miR-103 was differentially expressed in lung cancer (Garofalo *et al.* 2012), colorectal cancer (Chen *et al.* 2012), human gastric carcinoma (Zhang *et al.* 2015), and breast cancer (Kleivi Sahlberg *et al.* 2015). Although, miR-103 has been investigated extensively in mammalian diseases, studies on gga-miR-103 in chickens are still limited. In recent years, only a few studies on chicken gga-miR-103 have been reported, including deep sequencing in chicken preadipocytes (Yao *et al.* 2011), hypothalamus tissue (Sun *et al.* 2012), chromium metabolism (Pan *et al.* 2013), abdominal fatness (Wang *et al.* 2015), and abdominal adipose tissues (Huang *et al.* 2015). As for MD, it was reported that gga-miR-103-3p was expressed differentially between MDV-infected and uninfected CEF cells (Burnside *et al.* 2008). In MDV-infected chickens of line 7_2_ (MD susceptible line), the expression of gga-miR-103 was significantly downregulated compared with noninfected chickens at 21 dpi (Tian *et al.* 2012). Our results showed that gga-miR-103-3p was greatly decreased in MDV-infected tissues, which is consistent with the previous studies (Burnside *et al.* 2008; Tian *et al.* 2012).

Several studies have shown that miR-103 is involved in cancers, and in regulated cell proliferation and migration. A vital molecular relationship between miR-103 and *PER3* (period circadian clock 3) in human colorectal cancer (CRC) cell lines was confirmed. The proliferation and migration of CRC cells could be regulated by miR-103 (Hong *et al.* 2014). MiR-103/107 overexpression or *CDK5R1* [cyclin-dependent kinase 5, regulatory subunit 1 (p35)] silencing caused a reduction in SK-N-BE migration ability (Moncini *et al.* 2011). Our results suggested that gga-miR-103-3p suppressed cell migration in the MDCC-MSB1 cell line.

miRNAs play critical roles in gene regulatory networks. An individual miRNA can target hundreds or thousands of different mRNAs; meanwhile, an individual gene can be coordinately suppressed by multiple various miRNAs (Krek *et al.* 2005; Friedman *et al.* 2009). The miR-103 family has been found in 23 species (Griffiths-Jones 2004; Griffiths-Jones *et al.* 2006). A total of 257 conserved sites, and 79 poorly conserved sites, were predicted using TargetScan, and the seed regions, which aligned to mRNA 3ʹ-UTR of targets, were the same in different species, suggesting that the targets were highly conserved among human, chicken, and some other species (http://www.targetscan.org/cgi-bin/targetscan/vert_61/targetscan.cgi?mirg=gga-miR-103). Considering the results of TargetScan, miRDB, and GO analysis, we selected two target genes, *CCNE1* and *TFDP2*, for further validation. The product of the *CCNE1* gene forms a complex with CDK2 (cyclin-dependent kinase 2), and functions as a regulatory subunit, which is required for cell cycle G1/S transition (Ekholm and Reed 2000; Mazumder *et al.* 2004; Sherr 2000; Sherr and Roberts 1995). Overexpression of this gene can cause chromosome instability in many tumors; thus, it may contribute to tumorigenesis. The expression level of *CCNE1* begins to rise in mid-G1 phase, peaks during late G1, and declines around the G1/S transition. So the timing of its expression level plays a critical role in initiation of DNA replication as well as chromatin remodeling during tumorigenesis (Hu *et al.* 2014). *CCNE1* is the target gene of miR-15/16 in human lung cancer (Bandi and Vassella 2011) and glioblastoma (Xia *et al.* 2009) during tumorigenesis (http://www.kegg.jp/kegg-bin/show_pathway?hsa05206+898). From the point of view of cancer or tumor research, it was reported that miR-103 is part of the G1/S transition regulatory network, by targeting *CCNE1*, *CDK2*, and *CREB1* (cAMP responsive element binding protein 1) during IGF-1 simulated proliferation in mouse crypt cells (Liao and Lonnerdal 2010). miR-107, as a paralog of miR-103, was identified in human nonsmall cell lung cancer, and the potential target *CCNE1* was downregulated by transfection with miR-107 (Takahashi *et al.* 2009). In this study, we identified a binding site of gga-miR-103-3p in the 3′UTR of *CCNE1*, and verified that *CCNE1* was a target gene of gga-miR-103-3p.

The *TFDP2* gene (E2F/DP2) belongs to the transcription factor dimerization partner (DP) family, and its encoded protein forms heterodimers with the E2F transcription factor, resulting in transcriptional activation of cell cycle regulated genes (Wu *et al.* 1995; Zhang *et al.* 1997). E2F complexes can be broadly classified into two subgroups: “activating” E2Fs (E2F1, E2F2, and E2F3), and “repressive” E2Fs (E2F4, E2F5, and E2F6) (Trimarchi and Lees 2002). Studies have suggested that E2F1 is the target gene of miR-20a (Sylvestre *et al.* 2007), miR-106b (Ambs *et al.* 2008), and miR-330 (Lee *et al.* 2009) in prostate cancer. These two target genes are involved in the cell cycle pathway (KEGG Pathway: map 04110), and affect cell cycle progression from G1 phase to S phase. Therefore, aberrant expressions of these two genes may disturb the normal cell cycle, and further cause tumorigenesis (http://www.genome.jp/kegg-bin/show_pathway?map04110). Meanwhile, the Cyclin E family and E2F factors participate in the pathways in cancer (KEGG Pathway: ko 05200), and can affect cell proliferation indirectly (http://www.genome.jp/kegg-bin/show_pathway?map05200). Additionally, *TFDP2* could be targeted by miR-122, which inhibits *c-Myc* transcription in hepatocellular cancer indirectly (B. Wang *et al.* 2014). Here, we verified that TFDP2 was another target gene of gga-miR-103-3p by luciferase reporter assay and western blot assay.

In summary, we found that gga-miR-103-3p was downregulated in MDV-infected tissues, and it inhibits cell migration in MDCC-MSB1 cell lines. The two target genes *CCNE1* and *TPDP2* were identified. The effective binding sites of gga-miR-103-3p in the 3ʹ-UTR of *CCNE1* and *TFDP2* mRNA were confirmed. Gga-miR-103-3p could suppress *CCNE1* gene expression at both mRNA and protein levels, while the *TPDP2* gene was regulated by gga-miR-103-3p at the protein but not mRNA level. Gga-miR-103-3p, working together with its targets, may play a potential role in MDV tumorigenesis.

## Supplementary Material

Supplemental Material
