# Supplementary material for: Chicken gga-miR-103-3p Targets CCNE1 and TFDP2 and Inhibits MDCC-MSB1 Cell Migration
Source: G3 (Bethesda). 2016 Mar 1;6(5):1277–85. doi: 10.1534/g3.116.028498 (PMC4856079; doi:10.1534/g3.116.028498)
Supplement: Supplemental Material [file supp_6_5_1277__index.html]

Chicken gga-miR-103-3p Targets CCNE1 and TFDP2 and Inhibits MDCC-MSB1 Cell Migration — Supplemental Material 

# Chicken gga-miR-103-3p Targets CCNE1 and TFDP2 and Inhibits MDCC-MSB1 Cell Migration

## Supplemental Material for Han *et al.*, 2016

**Files in this Data Supplement:**

- File S1 - The candidate target genes of gga-miR-103-3p predicted by TargetScan and miRDB, and clustered by GO analysis. (.xls, 932 KB)
- Figure S1 - The schematic of experimental design. NC: negative control. (.tif, 4 MB)
